# Long-Term Results of Pars Plana Vitrectomy with Internal Limiting Membrane Peeling for Vitreomacular Traction Syndrome: A Prospective Investigation in Central Asian Population

**DOI:** 10.3390/healthcare13010044

**Published:** 2024-12-30

**Authors:** Gulnar Zhurgumbayeva, Dastan Kyrykbayev, Kairat Ruslanuly, Susanne Binder, Mukhit Kulmaganbetov

**Affiliations:** 1 Kazakh Eye Research Institute, Almaty A05H2A8, Kazakhstand.kyrykbaev@eyeinst.kz (D.K.);; 2 Department of Ophthalmology, Sigmund Freud University, 1020 Vienna, Austria; susanne@susannebinder.com; 3 Department of Ophthalmology, Weill Cornell Medicine, New York, NY 10065, USA; 4 Centre for Eye and Vision Research (CEVR), Hong Kong

**Keywords:** vitreomacular traction syndrome, lamellar macular hole, full-thickness macular hole, pars plana vitrectomy, Central Asia

## Abstract

**Background:** There is a lack of research on the clinical characteristics of vitreomacular traction (VMT) in the Central Asian population, which evaluates the visual recovery and macular hole closure outcomes of pars plana vitrectomy (PPV) with membrane peel in this population. **Methods:** This long-term prospective cohort study, conducted at the Kazakh Eye Research Institute from June 2015 to December 2021 with a follow-up period until December 2022, included 1574 patients (1784 eyes) with VMT syndrome. Among the eyes, 724 (40.58%) had VMT, 620 (34.75%) had a lamellar macular hole (LMH), and 440 (24.66%) had a full-thickness macular hole (FTMH). **Results:** The FTMH group consisted of small (≤250 μm) holes in 14 (3.2%) eyes, medium (250–400 μm) holes in 79 (17.9%) eyes, and large (>400 μm) holes in 347 (78.9%) eyes. Significant improvements in visual acuity, retinal thickness reduction, and macular hole closure were observed in 98.79% of patients with LMH and 81.14% of patients with FTMH at 1.5 months after surgery. PPV with membrane peel resulted in improved clinical outcomes, including enhanced visual acuity and macular structure. **Conclusions:** These findings support the existing research indicating the efficacy and relative safety of this surgical approach for VMT, despite the potential risks of postoperative complications.

## 1. Introduction

Vitreomacular traction syndrome (VMTS) is a condition characterized by the persistence of vitreous attachment to the macula, resulting in functional impairment [1,2,3,4]. The estimated prevalence of VMTS is 1365 cases per 100,000 population, with an annual incidence rate of 6.96 new cases per 100,000 population [1,2,3,4]. In some cases, VMT can progress to a more advanced stage, leading to the development of a full-thickness macular hole (FTMH) and irreversible visual loss [2,3,5,6].

Macular hole (MH) is a specific manifestation of VMTS and is defined as a defect in the retinal tissue within the foveal zone, accompanied by cystic retinal changes and neuroepithelial detachment [1,7]. Surgical intervention is often necessary to address VMTS and pars plana vitrectomy (PPV) with an internal limiting membrane (ILM) peeling has been recognized as an effective approach for relieving tractional forces and stabilizing the retinal surface, leading to visual function recovery [1,8,9,10,11].

Despite the widespread recognition of PPV with ILM peeling as the most effective treatment for VMTS, there is a noticeable gap in research focusing on the clinical features of VMT in the Central Asian population. To address this gap, we conducted a long-term prospective study at Kazakhstan’s national referral center to evaluate the effectiveness of PPV with ILM peel in terms of visual recovery and closure of MH.

The primary objective of our 7-year prospective study was to evaluate the clinical characteristics and surgical outcomes of PPV with ILM peeling in patients with VMTS in the Central Asian population. Specifically, we aimed to (1) assess pre- and post-surgical best-corrected visual acuity (BCVA) and central retinal thickness (CRT) by comparing these parameters before surgery and on the 6th day after surgery to determine the immediate impact of the intervention; (2) evaluate long-term outcomes by analyzing the long-term follow-up data on BCVA and CRT to assess the sustained effects of the surgical intervention over an extended period (up to 12 months); and (3) provide insights into the clinical features and outcomes of VMTS in the Central Asian population, thereby contributing to the global understanding of the disease and its management.

By achieving these objectives, our study aims to fill the existing research gap and improve the management and treatment outcomes for individuals with VMTS in our region.

## 2. Materials and Methods

This single-center, prospective cohort study was conducted at a tertiary eye care facility, Kazakh Eye Research Institute (Almaty, Kazakhstan), from June 2015 to December 2021, and all patients diagnosed with VMTS during this period were enrolled in this study. The follow-up period extended until 30 December 2022. This study was approved by the Kazakh Eye Research Institute’s Local Ethics Committee (No. 01-2015 on 18 February 2015) and adhered to the Declaration of Helsinki’s principles.

### 2.1. Vitreomacular Traction Syndrome Classification

The classification of vitreomacular traction syndrome (VMTS) in this study was based on the established criteria provided by the International Vitreomacular Traction Study Group [1]: (1) vitreomacular traction (VMT), characterized by abnormal posterior vitreous detachment leading to foveal distortion; (2) lamellar macular hole (LMH), characterized by partial thickness discontinuity in the foveal neurosensory retina while maintaining intact outer retinal layers; and (3) full-thickness macular hole (FTMH), which involves complete disruption of all retinal layers within the fovea.

### 2.2. Participants

Eighteen years or older patients with a confirmed diagnosis of VMTS based on clinical examination and optical coherence tomography (OCT) findings who met the defined inclusion criteria, which also included a preoperative visual acuity of counting fingers or better [12] and suitable candidates for PPV with ILM peeling, were invited to participate in this study. Patients must be residents of the Central Asian region to ensure this study population is representative of this demographic. Informed consent was obtained either directly from the patients or through their legal representative(s) prior to enrollment.

Exclusion criteria encompassed individuals with a preoperative visual acuity of light perception or worse, as well as those who were unable to undergo preoperative and immediate postoperative OCT assessments due to conditions such as dense cataracts or corneal opacity, resulting in opaque media. Patients who had undergone previous vitrectomy or other significant ocular surgeries in this study eye, with systemic conditions that could affect study participation or outcomes (uncontrolled diabetes or severe cardiovascular disease), were excluded. Additionally, patients who did not attend the post-surgical 6-day follow-up were excluded from this study.

### 2.3. Preoperative and Postoperative Evaluations

A comprehensive ophthalmologic examination was performed both before surgery and on the 6th postoperative day. Autorefractometry was conducted to determine the refractive error, while unaided visual acuity and BCVA were measured using a Snellen chart presented in LogMAR format. Slit-lamp biomicroscopy was utilized to examine the anterior segment of the eye, followed by a dilated fundus examination (DFE) using tropicamide 1% to visualize the posterior segment. A mandatory DFE was performed two weeks prior to surgery and one month after surgery, with retinal laser photocoagulation administered if retinal breaks or tears were detected.

Macular assessments and measurements of CRT were carried out using OCT. Ocular biometry, specifically axial length measurement, was obtained using swept-source OCT. In cases where axial length measurement was not feasible, immersion ultrasound A-scan was employed as an alternative method.

In addition to the pre- and post-surgical assessments, additional optional follow-up visits at 1.5, 3, 6, and 12 months after surgery were strongly recommended. If data from these visits were collected, they were included in the analysis.

### 2.4. Surgery and Follow-Up

Patients diagnosed with VMT and LMH underwent a comprehensive monitoring process for three months before considering surgical intervention. During this observation period, various factors were taken into account, including a decline in visual acuity (0.54–0.30 LogMAR or worse), the presence of visual distortion (metamorphopsia), and notable changes in the macular profile indicating an increased risk of developing an idiopathic FTMH.

All patients underwent PPV with ILM peel procedure, which was performed by the same surgeon (G.Zh.). The surgical equipment utilized was the ophthalmic microsurgery system Constellation Vision System (Alcon, Geneva, Switzerland), employing a frequency of 5000–10,000 cuts/minute and a vacuum level of up to 650 mmHg. ILM staining was conducted using trypan blue ophthalmic solution 0.15% (MembraneBlue, DORC, Zuidland, The Netherlands), and the stained ILM was carefully removed using Alcon Grieshaber 25GA+ and Alcon Grieshaber 27GA+ forceps (Switzerland). The surgery concluded with a fluid-gas exchange, replacing the vitreous gel with a gas bubble to provide support and facilitate the closure of the MH.

Both LMH and FTMH cases underwent similar vitrectomy and fluid-gas exchange procedures. However, the distinction in surgical steps was observed during membrane peeling, with ILM peeling performed over the lamellar hole area for patients with LMH and over the entire macular hole area for patients with FTMH.

In cases of visually significant cataracts, additional cataract removal surgery was performed using phacoemulsification with intraocular lens (IOL) implantation. Following the surgical procedures, all patients were instructed to maintain a face-down position for three days and received combined antibacterial and anti-inflammatory eye drops for one month.

### 2.5. Statistical Analysis

Statistical analysis was performed by two independent researchers using GraphPad Prism (Version 3.0, GraphPad Software Inc., San Diego, CA, USA) and SPSS (Version 29.0.0, IBM SPSS Statistics, Chicago, IL, USA) software programs to ensure impartiality and minimize biases. Continuous data were summarized as mean ± standard deviation (SD) for normally distributed variables and as median and interquartile range (IQR) or range for non-normally distributed variables. Nonparametric tests, including the Wilcoxon signed-rank test and the Mann–Whitney U test, were used for data analysis. A *p*-value of <0.05 was considered statistically significant.

## 3. Results

**Figure 1:**
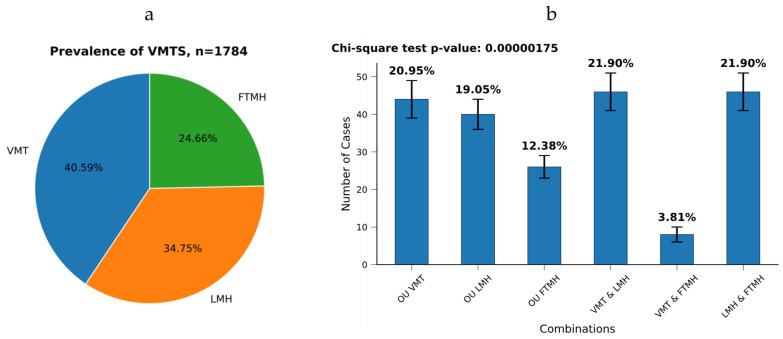
Prevalence of vitreomacular traction syndrome (VMTS) within this study population (**a**) and their combinations (**b**). Abbrev.: VMT, vitreomacular traction; LMH, lamellar macular hole; FTMH, full-thickness macular hole; OU—both eyes.

A total of 1574 patients diagnosed with VMTS (1784 eyes) were prospectively analyzed in this study (Table 1). The mean age of the patients was 68.39 ± 8.94 years (range: 18 to 90 years). The majority of the patients were female, accounting for 1228 (78.02%) of the cases, while 346 (21.98%) were male (*p* > 0.001). Upon evaluation, VMT was found in 724 eyes, LMH in 620 eyes, and FTMH in 440 eyes (Figure 1a). Figure 1b depicts the various combinations of VMTS (n = 210) observed in patients with both symmetrical and asymmetrical binocular VMTS (*χ*^2^ < 0.05).

Age-related macular degeneration (AMD) was detected in 83% of patients, making it the most prevalent concomitant ocular condition. Cataract was diagnosed in 76.5% of cases, followed by hyperopia in 37.5% and myopia in 33.2% of the cases. A summary of the identified concomitant ocular diseases is provided in Table 2. The distribution of concomitant diagnoses significantly differs from the expected distribution (*p*-value < 0.05).

A total of 1776 eyes (99.55%) underwent a fluid-air exchange procedure as part of the surgical intervention. Silicone oil tamponade was performed in 8 eyes (0.45%), with 6 eyes presenting proliferative diabetic retinopathy, 1 eye experiencing intraoperative vitreous due to post-thrombotic retinopathy, and 1 eye with iatrogenic FTMH measuring 702 μm in diameter. Eyes with proliferative diabetic retinopathy and post-thrombotic retinopathy also received retinal laser photocoagulation. Furthermore, 1365 eyes (76.51%) required phacoemulsification of cataracts with IOL implantation. In cases of lens subluxation, a capsular tension ring was installed. In one instance, the removal of a dislocated IOL was followed by secondary IOL implantation with transscleral fixation.

**Figure 2:**
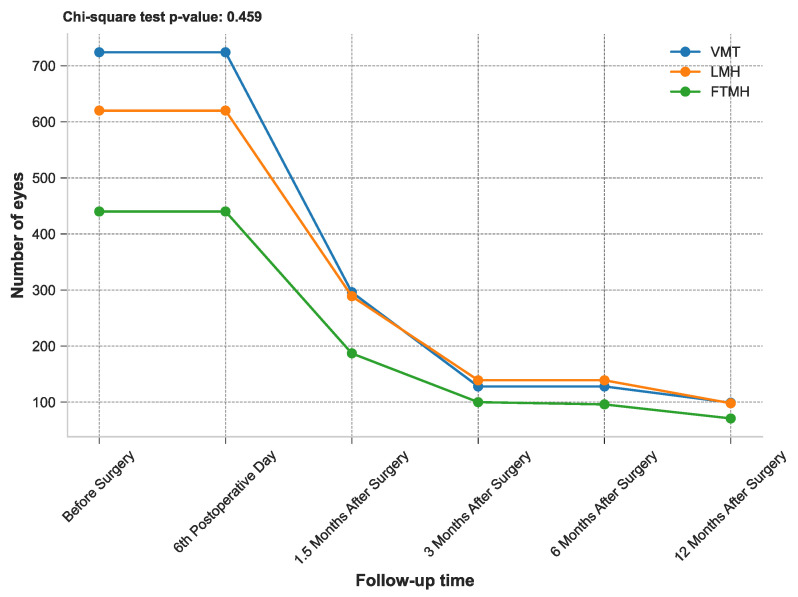
Study timeline and number of total eyes attending the follow-up check.

Figure 2 depicts this study timeline and the total number of eyes attending the follow-up check. Notably, it is important to highlight that patient participation in these follow-up visits was voluntary and not obligatory. Nevertheless, a subset of patients opted to continue their visits to the clinical setting. The high *χ*^2^ value suggests that there are significant differences in the number of eyes observed at various follow-up times, indicating that the progression or resolution of conditions like VMT, LMH, and FTMH over time is not uniform and may reflect meaningful clinical changes post-surgery.

All patients (n = 1784 eyes) continued to participate in the follow-up check on the 6th postoperative day. However, the number of eyes attending the further long-term follow-up assessments gradually decreased at subsequent time points, with 772 eyes at 1.5 months after surgery, 367 eyes at 3 months after surgery, 363 eyes at 6 months after surgery, and 268 eyes at 12 months after surgery.

### 3.1. Vitreomacular Traction

**Figure 3:**
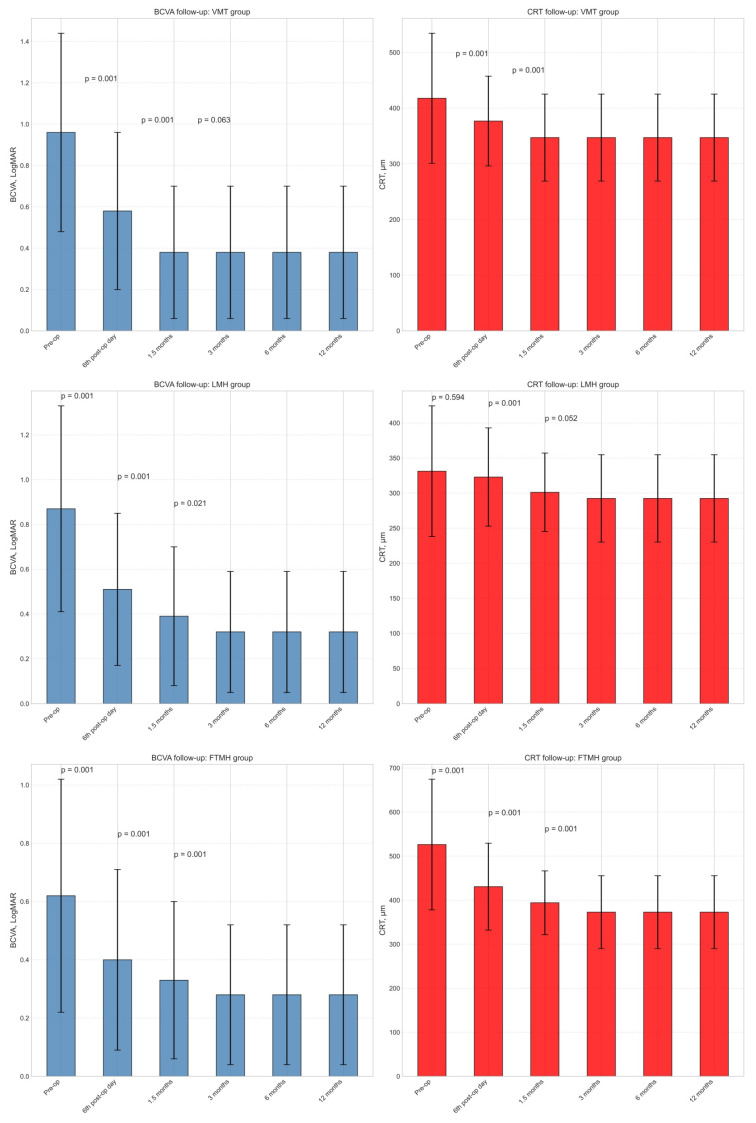
Change in the best-corrected visual acuity (BCVA, **left column**) and central retinal thickness (CRT, **right column**) after surgery on day 6, months 1.5, 3, 6, and 12 in patients with pre-surgical vitreomacular traction (VMT, **top row**), lamellar macular hole (LMH, **middle row**), and full-thickness macular hole (FTMH, **bottom row**).

In the VMT group, we observed a significant improvement in mean BCVA (Figure 3, top left) from 0.96 ± 0.48 LogMAR before surgery to 0.58 ± 0.38 LogMAR on the 6th postoperative day and further to 0.38 ± 0.32 LogMAR after 1.5 months (*p* < 0.001 for both time points). We also noticed a trend towards statistical significance at 3 months postoperatively (*p* = 0.063). However, no significant changes in visual function were observed at the follow-up examinations from 6 months to 1 year postoperatively.

Regarding the mean CRT in the VMT group (Figure 3, top right), we found a significant decrease from 417.7 ± 116.8 μm before surgery to 376.8 ± 80.6 μm at the 6th postoperative day and further to 347.1 ± 78.1 μm after 1.5 months (*p* < 0.001 for both time points). However, no significant changes in CRT were observed during the follow-up examinations from 3 months to 1 year. More detailed results on BCVA and CRT follow-up are shown in Supplementary Table S1.

### 3.2. Lamellar Macular Hole

In the LMH group, we observed a significant improvement in mean BCVA up to 3 months after surgery (Figure 3, middle left, and Supplementary Table S2). The mean BCVA improved from 0.87 ± 0.46 LogMAR before surgery to 0.51 ± 0.34 LogMAR on the 6th postoperative day, further to 0.39 ± 0.31 LogMAR after 1.5 months, and finally to 0.32 ± 0.27 LogMAR after 3 months (*p* < 0.001, <0.001, and =0.021, respectively). However, no significant changes in visual function were observed during the follow-up examinations from 3 months to 1 year postoperatively.

Additionally, we found that the mean CRT decreased from 331.2 ± 93.2 μm before surgery to 322.9 ± 70.1 μm on the 6th postoperative day (Figure 3, middle right). It further decreased to 301.1 ± 56.0 μm after 1.5 months and 292.4 ± 62.3 μm after 3 months (*p* = 0.594, <0.001, and = 0.052, respectively).

### 3.3. Full-Thickness Macular Hole

The distribution of FTMH according to size was as follows: small (≤250 μm)—14 cases (3.2%), medium (250–400 μm)—79 cases (17.9%), large (>400 μm)—347 cases (78.9%).

In the FTMH group, a significant improvement in mean BCVA was observed from 0.62 ± 0.40 LogMAR preoperatively to 0.40 ± 0.31 LogMAR on the 6th postoperative day (Figure 3, bottom left), 0.33 ± 0.27 LogMAR after 1.5 months, and 0.28 ± 0.24 LogMAR after 3 months (*p* < 0.001 in all cases). No changes in visual function were observed at follow-up examinations from 3 months to 1 year postoperatively.

Significant decreases in mean CRT were observed in the FTMH group, from 526.3 ± 148.3 μm preoperatively to 430.7 ± 98.7 μm on the 6th postoperative day (Figure 3, bottom right, and Supplementary Table S3), 394.1 ± 72.4 μm after 1.5 months, and 372.8 ± 82.7 μm after 3 months (*p* < 0.001 in all cases). No changes in CRT were observed at follow-up examinations from 3 months to 1 year.

**Figure 4:**
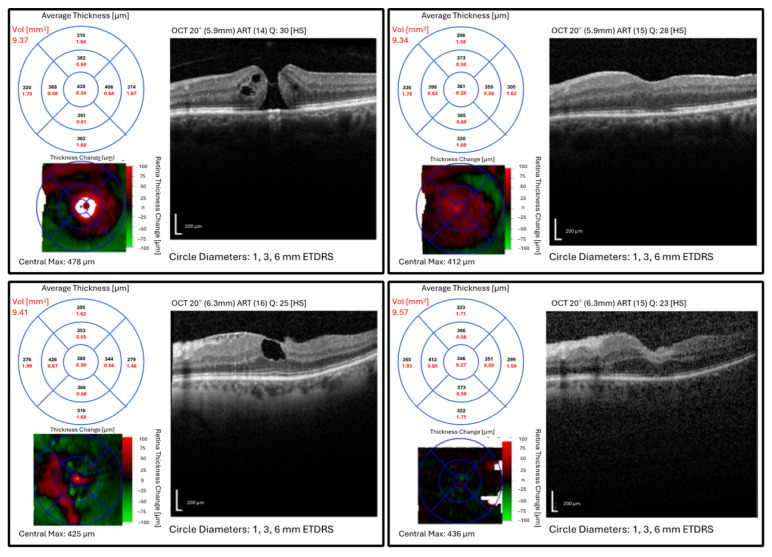
Macular optical coherence tomography images, average thickness and volume within ETDRS grid (1, 3, and 6 mm), and thickness change map of patients with a full-thickness macular hole before (**top left**) and after (**top right**) surgery and lamellar macular hole before (**bottom left**) and after (**bottom right**) surgery. Abbrev.: ETDRS, Early Treatment Diabetic Retinopathy Study; Vol, volume.

**Figure 5:**
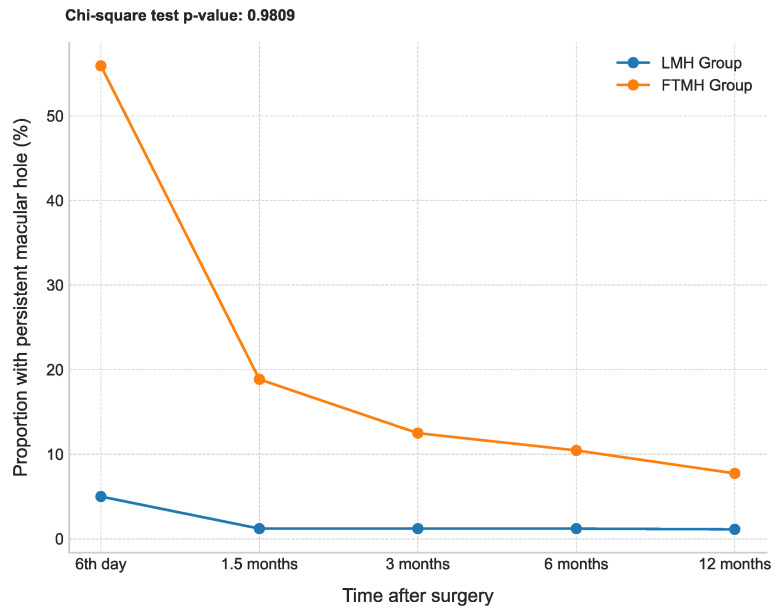
Patients with persistent macular holes after surgery at different time points. Abbrev.: LMH, lamellar macular hole; FTMH, full-thickness macular hole.

Regardless of the surgical intervention (Figure 4), there were patients with persistent MH after the surgery (Figure 5). An initial proportion of 5% exhibited persistent MH on the 6th postoperative day in the cohort of patients with LMH. This percentage decreased to 1.2% at 1.5 months after surgery and remained constant at 3, 6, and 12 months after the procedure. This decrease could be attributed to the loss of follow-up, where patients with persistent MHs did not return for subsequent evaluations. Additionally, some MHs may have closed spontaneously due to delayed healing processes, or variations in surgical technique and postoperative care might have influenced the outcomes.

Conversely, among the patients with FTMH, there was a substantially higher proportion of individuals with persistent MH immediately after surgery, with 55.91% still exhibiting this condition on the 6th postoperative day. Over time, a gradual decline in the proportion of persistent MH was observed, with percentages of 18.86%, 12.5%, 10.45%, and 7.73% at 1.5 months, 3 months, 6 months, and 12 months after surgery, respectively. The *χ*^2^ = 0.98 suggests that there are significant differences in the proportions of persistent MHs observed at various follow-up times, indicating that the likelihood of persistence decreases significantly over time for both the LMH and FTMH groups.

These findings indicate that the FTMH group initially displayed a greater susceptibility to persistent MH following surgery in comparison to the LMH group. However, as time progressed, both groups demonstrated a decline in the proportion of persistent MH, suggesting a degree of improvement or closure of the MHs. Supporting the above, Table 3 shows the MH diameter visualized using OCT after the surgery in both groups.

In terms of complications, 118 eyes (6.62%) experienced a postoperative increase in intraocular pressure (IOP), which was managed with topical antiglaucoma medications. One eye developed a retinal detachment that required surgical repair with vitrectomy and silicone oil tamponade. One eye experienced a recurrence of FTMH, which was treated with repeat vitrectomy and gas tamponade.

## 4. Discussion

This study aimed to investigate the outcomes of surgical intervention for Central Asian patients with VMTS, encompassing VMT, LMH, and FTMH. This study highlights the effectiveness of PPV with ILM peeling in improving vision and closing macular holes. Significant improvements in vision and CRT were observed post-surgery for patients with VMTS, with the most significant enhancements seen in the early postoperative phase. However, no substantial changes were noted beyond 6 months postoperatively, indicating limited long-term durability. Similar findings were reported by Blautain et al. (2022) during an 18-month follow-up after ILM peeling [13].

In patients with LMH, notable improvements in clinical outcomes were observed up to 3 months post-surgery. Wang and Wang (2020) demonstrated enhanced visual and anatomical outcomes in LMH cases associated with highly myopic foveoschisis following ILM peeling with gas tamponade [14]. In our study, significant changes beyond 3 months post-surgery were not observed. For FTMH, significant improvements in BCVA and CRT were observed after PPV+ILM peeling, with no significant changes during the follow-up period, underscoring the efficacy of surgical intervention in enhancing visual function in patients with FTMH.

Despite the high rates of MH closure and improved vision post-surgery, the puzzling issue remains as to why certain patients do not experience vision improvement despite successful surgery and macular structure restoration. This prompts the consideration of additional approaches for vision restoration after surgery, such as adjuvant pharmacological treatments [15], visual rehabilitation programs, genetic and molecular interventions [16], and the incorporation of novel surgical techniques in VMTS management [17]. Exploring these supplementary approaches may unveil new strategies to enhance vision restoration and improve outcomes for patients with VMTS who may not derive full benefit from standard surgical interventions alone. This comprehensive patient-centered approach could lead to more personalized and effective treatment strategies in the future.

Several surgical options that could be considered for patients with VMTS include techniques involving inverted ILM flaps [18], macular hydrodissection [19], and the transplantation of an amniotic membrane graft [20] or autologous retinal tissue [21]. The selection of the appropriate technique for each patient, taking into account baseline characteristics, disease duration, and MH size, should be refined according to the CLOSE [17] and MHCP [22] classifications. While the use of intravitreal Ocriplasmin before proceeding with PPV may be deemed a suitable and safe approach for treating vitreomacular adhesion [23], this recombinant protease is not approved in Kazakhstan and, thus, cannot be utilized in the clinical setting.

Post-surgical visual function assessment could also involve perimetry and microperimetry. However, as the indices of these methods correlate with BCVA [24], their use in a large cohort was not considered. This study only assessed OCT outcomes by monitoring CRT and MH size values, although the analysis of internal retinal layers with a machine learning approach could also be employed to evaluate the viability of subtle retinal neurons [25,26].

While face-down positioning was advised for three days for all patients, Ye et al. (2019) suggested that this may not be necessary for small MHs (>400 μm) [27]. Additionally, Zhang et al. (2019) found that face-down positioning did not delay MH closure or decrease the closure rate [28]. Mandatory follow-up assessments after the 6th day were conducted to monitor post-operative endophthalmitis incidence and MH recurrence and evaluate refractive correction. Subsequent follow-up visits were deemed optional, considering the patients’ residence in remote regions of Kazakhstan.

The decision to perform surgery for VMT and LMH hinges on various factors, including the morphological features of the condition and the presence of visual symptoms [1,29]. Tractional LMH often necessitates surgical intervention to alleviate traction and enhance outcomes, while the decision for degenerative LMH is contingent on the presence of vascular abnormalities and associated visual symptoms [30,31].

The results of this study contribute to the existing literature by offering insights into the clinical characteristics and outcomes of VMTS in the Central Asian population. This study’s focus on evaluating the effectiveness of PPV with ILM peeling in patients with VMTS was driven by its recognized efficacy in alleviating tractional forces and stabilizing the retinal surface, leading to visual function improvement. The choice of ILM peeling was grounded in its proven effectiveness in addressing vitreomacular traction and macular hole formation [32].

Understanding how demographic characteristics and disease duration influence treatment response can aid in refining patient selection and enhancing overall management strategies for VMTS across diverse populations. This study underscores the importance of close monitoring and early intervention in patients with AMD to forestall VMTS development or progression, given the high prevalence of VMTS in AMD patients [33,34]. Additionally, considering cataract surgery as a concurrent procedure during VMTS surgical management is advised due to the high incidence of cataracts in this patient cohort [35,36].

## 5. Conclusions

This study sheds light on the outcomes of surgical interventions for Central Asian patients with VMTS, specifically focusing on VMT, LMH, and FTMH. The effectiveness of PPV with ILM peeling in improving vision and closing macular holes was demonstrated, with significant improvements observed in BCVA and CRT post-surgery. Future research should continue to explore new approaches, refine patient selection criteria, and enhance management strategies to optimize outcomes for patients with VMTS in diverse populations.

## Figures and Tables

**Table 1:** Summary of demographic data.

Indicator	VMTS (n_px_ = 1574; n_eye_ = 1784)			*p*-Value
	VMT	LMH	FTMH	
Age (mean ± SD)	67.28 ± 10.10	69.34 ± 9.30	68.38 ± 9.50	0.09
Gender				>0.001
Male	185	94	67	
Female	673	351	204	

**Table 2:** Concomitant ocular conditions diagnosed in this study’s participants.

Concomitant Diagnosis	Case Number	%	*χ^2^* Test *p*-Value
Age-related macular degeneration	1481	83	0.00073
Cataract	1365	76.5	
Hyperopia	670	37.5	
-low: +2.00D or less	346	51.6	
-moderate: +2.25D to +5.00D	286	42.7	
-high: +5.00D or higher	38	5.7	
Myopia	592	33.2	
-mild: 0.00D to −1.50D	258	43.6	
-moderate: −1.50D to 6.00D	243	41	
-high: −6.00D or more	91	15.3	
Peripheral retinal degeneration	427	23.9	
Glaucoma	401	22.5	
Pseudophakia	398	22.3	
Diabetic retinopathy	73	4	
-non-proliferative stage	16	22	
-pre-proliferative stage	35	47.9	
-proliferative stage	22	30.1	
Post-thrombotic retinopathy	35	2	
Vitreous body clouding—synchysis scintillans	35	2	
Hemophthalmos	13	0.7	
Traumatic central serous chorioretinopathy	11	0.6	
Retinitis pigmentosa	8	0.43	
Optic nerve atrophy	7	0.4	
Lens subluxation	3	0.2	
Intraocular lens dislocation to the vitreous body	1	0.05	

**Table 3:** Parameters of the persistent macular hole after surgery.

Timeline of the Follow-Up Check	Macular Hole Parameters, μm (Median; Range)		t-Statistic *p*-Value
	LMH	FTMH	
Before surgery	396.8 ± 123.3 (365; 120–1168)	625.1 ± 293.1 (577.5; 137–2810)	0.0002
6th postoperative day	389.0 ± 290.2 (403; 10–1242)	503.3 ± 293.9 (444; 60–2180)	0.0062
1.5 months after surgery	265.0 ± 148.6 (258; 120–417)	625.4 ± 284.9 (636; 140–1504)	<0.001
3 months after surgery	418.8 ± 214.8 (402.5; 200–808)	698.9 ± 309.4 (690; 188–2058)	<0.001
6 months after surgery	384.9 ± 222.2 (345; 100–738)	663.0 ± 366.9 (582; 80–1943)	<0.001
12 months after surgery	331.4 ± 185.1 (400; 100–500)	759.6 ± 292.0 (749.5; 250–1673)	<0.001

## Data Availability

The data that support the findings of this study are available on request from the corresponding author. The data are not publicly available due to privacy or ethical restrictions.

## Supplementary Materials

The following supporting information can be downloaded at: https://www.mdpi.com/article/10.3390/healthcare13010044/s1; Table S1: BCVA and OCT-based CRT results among VMT group’s patients before and after surgery; Table S2: BCVA and OCT-based CRT results among LMH group’s patients before and after surgery; Table S3: BCVA and OCT-based CRT results among FTMH group’s patients before and after surgery.
