# Supplementary material for: Long-Term Results of Pars Plana Vitrectomy with Internal Limiting Membrane Peeling for Vitreomacular Traction Syndrome: A Prospective Investigation in Central Asian Population
Source: Healthcare (Basel). 2024 Dec 30;13(1):44. doi: 10.3390/healthcare13010044 (PMC11720311; doi:10.3390/healthcare13010044)
Supplement: Supplementary file 1 [file healthcare-13-00044-s001.zip › healthcare-3284660-supplementary.pdf]

SUPPLEMENTARY MATERIALS

Tables

Table S1: BCVA and OCT-based CRT results among VMT group's patients before and after surgery

Timeline and number of total eyes attending the follow-up check	Mean ± SD (Median; Range)	
	BCVA, LogMAR	CRT, μm
Before surgery, n=724	0.96 ± 0.48 (0.82; 0.15 – 2.3)	417.7 ± 116.8 (403.5; 166 – 1015)
6th postoperative day, n=724	0.58 ± 0.38 (0.52; 0.0 – 2.3)	376.8 ± 80.6 (374; 123 – 765)
1.5 months after surgery, n=296	0.38 ± 0.32 (0.3; 0.0 – 2.3)	347.1 ± 78.1 (346; 95 – 745)
3 months after surgery, n=128	0.38 ± 0.32 (0.3; 0.0 – 2.3)	340.1 ± 75.5 (380.5; 162 – 736)
6 months after surgery, n=128	0.32 ± 0.28 (0.26; 0.0 – 1.69)	332.3 ± 62.6 (338; 166 – 541)
12 months after surgery, n=99	0.32 ± 0.28 (0.3; 0.0 – 1.15)	326.6 ± 54.29 (328; 220 – 596)

Abbrev.: VMT, vitreomacular traction; SD, standard deviation; BCVA, best corrected visual acuity; CRT, central retinal thickness; μm, micrometer

Table S2: BCVA and OCT-based CRT results among LMH group's patients before and after surgery

Timeline and number of total eyes attending the follow-up check	Mean ± SD (Median; Range)	
	BCVA, LogMAR	CRT, μm
Before surgery, n=620	0.87 ± 0.46 (0.82; 0.0 – 2.3)	331.2 ± 93.2 (315; 125 – 929)
6th postoperative day, n=620	0.51 ± 0.34 (0.39; 0.0 – 2.0)	322.9 ± 70.1 (313; 86 – 733)
1.5 months after surgery, n=289	0.39 ± 0.31 (0.30; 0.0 – 2.0)	301.1 ± 56.0 (300; 131 – 448)

3 months after surgery, n=139	0.32 ± 0.27<br>(0.22; 0.0 – 1.3)	292.4 ± 62.3<br>(286; 87 – 504)
6 months after surgery, n=139	0.35 ± 0.33<br>(0.22; 0.0 – 2.0)	292.3 ± 72.4<br>(288; 83 – 521)
12 months after surgery, n=98	0.32 ± 0.31<br>(0.22; 0.0 – 1.3)	279.7 ± 65.2<br>(274.5; 82 – 530)

Abbrev.: LMH, lamellar macular hole; SD, standard deviation; BCVA, best corrected visual acuity; CRT, central retinal thickness; µm, micrometer

Table S3: BCVA and OCT-based CRT results among FTMH group's patients before and after surgery

Timeline and number of total eyes attending the follow-up check	Mean ± SD (Median; Range)	
	BCVA, LogMAR	CRT, µm
Before surgery, n=440	1.33 ± 0.41<br>(1.3; 0.52 -2.3)	364.0 ± 133.9<br>(354; 94 - 2225)
6th postoperative day, n=440	0.91 ± 0.33<br>(0.82; 0.09 - 2.0)	311.3 ± 129.9<br>(299; 28 - 2223)
1.5 months after surgery, n=187	0.84 ± 0.42<br>(0.82; 0.09 - 2.0)	296.0 ± 115.8<br>(273; 30 - 736)
3 months after surgery, n=100	0.86 ± 0.44<br>(0.82; 0.09 - 2.0)	296.5 ± 133.7<br>(267; 24 - 954)
6 months after surgery, n=96	0.85 ± 0.49<br>(0.82; 0.04 - 2.0)	275.7 ± 101.5<br>(101.5; 37 - 600)
12 months after surgery, n=71	0.86 ± 0.51<br>(0.82; 0.04 - 2.0)	243.6 ± 91.9<br>(246; 42 - 455)

Abbrev.: SD, standard deviation; BCVA, best corrected visual acuity; CRT, central retinal thickness; µm, micrometer
